# Metabolic Plasticity Aids Amphotropism of Coxiella burnetii

**DOI:** 10.1128/IAI.00135-21

**Published:** 2021-11-16

**Authors:** Savannah E. Sanchez, Alan G. Goodman, Anders Omsland

**Affiliations:** a Paul G. Allen School for Global Health, College of Veterinary Medicine, Washington State University, Pullman, Washington, USA; b School of Molecular Biosciences, College of Veterinary Medicine, Washington State University, Pullman, Washington, USA; Yale University School of Medicine

**Keywords:** *Coxiella burnetii*, interstitial fluid, physiological medium, glycolysis, gluconeogenesis, metabolic plasticity, amphotropism, intracellular parasites, obligate, virulence

## Abstract

Coxiella burnetii, the causative agent of query (Q) fever in humans, is an obligate intracellular bacterium. C. burnetii can naturally infect a broad range of host organisms (e.g., mammals and arthropods) and cell types. This amphotropic nature of C. burnetii, in combination with its ability to utilize both glycolytic and gluconeogenic carbon sources, suggests that the pathogen relies on metabolic plasticity to replicate in nutritionally diverse intracellular environments. To test the significance of metabolic plasticity in C. burnetii host cell colonization, C. burnetii intracellular replication in seven distinct cell lines was compared between a metabolically competent parental strain and a mutant, *CbΔpckA*, unable to undergo gluconeogenesis. Both the parental strain and *Cb*Δ*pckA* mutant exhibited host cell-dependent infection phenotypes, which were influenced by alterations to host glycolytic or gluconeogenic substrate availability. Because the nutritional environment directly impacts host cell physiology, our analysis was extended to investigate the response of C. burnetii replication in mammalian host cells cultivated in a novel physiological medium based on the nutrient composition of mammalian interstitial fluid, interstitial fluid-modeled medium (IFmM). An infection model based on IFmM resulted in exacerbation of a replication defect exhibited by the *Cb*Δ*pckA* mutant in specific cell lines. The *Cb*Δ*pckA* mutant was also attenuated during infection of an animal host. Overall, the study underscores that gluconeogenic capacity aids C. burnetii amphotropism and that the amphotropic nature of C. burnetii should be considered when resolving virulence mechanisms in this pathogen.

## INTRODUCTION

Coxiella burnetii, the causative agent of query (Q) fever in humans and coxiellosis in other species (e.g., cattle, sheep, and goats), is a highly infectious obligate intracellular bacterium (1, 2). While most human infections with C. burnetii result in asymptomatic disease, severe complications including hepatitis and endocarditis can develop (3, 4). A recent Q fever outbreak in the Netherlands with >4,000 diagnosed human cases and epidemiological estimates of ∼40,000 infected individuals (5–7) illustrates the clinical potential of C. burnetii as a human pathogen.

C. burnetii naturally infects a broad range of host organisms, including domestic and wild mammals, reptiles, birds, and arthropods (3). Because inhalation is the primary route by which C. burnetii is transmitted (8), alveolar macrophages are considered the initial and potentially primary host cell target for C. burnetii within lung tissue (9). In animal models, C. burnetii also infects professional phagocytes of lung (10) and liver (11) tissue. In addition to phagocytes, biopsies and microscopic analysis of C. burnetii-infected tissue have identified bacteria in placental trophoblasts (12, 13) and adipocytes (14), as well as pulmonary fibroblasts and type I alveolar cells (10). Within host cells, the pathogen exclusively replicates in a phagolysosome-derived compartment referred to as the *Coxiella* containing vacuole (CCV) (8, 15).

Similar to other obligate intracellular bacteria, C. burnetii has undergone reductive evolution, resulting in a relatively small (∼2 Mb) genome (16), yet encodes a largely intact central metabolic machinery. The significance of amino acids in C. burnetii physiology is broad, including a role for glutamate as a primary carbon source based on biochemical data (17), a correlation between amino acid auxotrophy with an abundance of genes encoding amino acid permeases and transporters (16, 18), and a positive correlation between host autophagic activity and CCV development (19–22), with potential implications for pathogen replication (23). Recently, generation of a C. burnetii mutant (the *Cb*Δ*pckA* strain) unable to undergo gluconeogenesis demonstrated that C. burnetii exhibits metabolic plasticity in carbon metabolism via the use of glucose for biomass production (24). Because extracellular components are trafficked into the CCV via fluid-phase endocytosis (25), composition of the nutritional environment, including concentrations of gluconeogenic amino acids and glucose, can be expected to directly impact the access of C. burnetii to nutrients and thus intracellular replication.

In the present study, the role of metabolic plasticity in supporting amphotropism (i.e., the ability to infect a broad range of host organisms and/or cell types) of C. burnetii was assessed. Intracellular replication within four distinct mammalian- and three arthropod-derived cell lines was compared between a metabolically competent parental strain and the *Cb*Δ*pckA* mutant. Additionally, because the nutritional environment directly impacts host cell physiology—upon which pathogen fitness is intimately dependent—the analysis was extended to investigate C. burnetii intracellular replication within mammalian cells cultivated in a novel physiological medium that resembles the nutrient composition of mammalian interstitial fluid (IF), interstitial fluid-modeled medium (IFmM).

## RESULTS

### Development and composition of IFmM.

Tissue culture media were originally designed for ease of maintenance and optimal replication of immortalized cell lines. For many commercially available media, the chemical composition and/or concentrations of specific nutrients do not necessarily reflect physiological conditions. To observe C. burnetii intracellular replication under conditions more reflective of nutrient availability within host tissues, a novel cell culture medium modeled on mammalian IF, IFmM, was designed. Mammalian IF serves as a conduit for supply of nutrients to tissues and thus is a physiologically relevant source of nutrients for most *Coxiella*-mammalian host interactions. In IFmM, concentrations of the principal ions sodium (Na^+^), potassium (K^+^), and chloride (Cl^−^) were based on those used in the commercially available medium RPMI 1640 (RPMI) (26) (see Table S1 in the supplemental material) and reported for IF (27, 28) (see Table S2 in the supplemental material). While commercial tissue culture media contain combinations of amino acids at concentrations that generally do not reflect physiological concentrations (Table S1), IFmM was designed to include all 20 amino acids at concentrations measured in IF (29, 30) (Table S2). Glucose concentrations in IF extracted from adipose or skeletal muscle tissues are reported to range from 2.0 to 4.5 and 2.1 to 4.8 mM, respectively (29); however, RPMI (26), Dulbecco modified Eagle medium (DMEM), and minimal essential medium (MEM) (31, 32) formulations contain glucose in excess at 11 and 5.5 mM, respectively (Table S1). To better reflect physiological conditions, the concentration of glucose in IFmM is 2.5 mM. Lastly, the availability and concentration of vitamins in IFmM are based on those found in RPMI (26). The complete composition of IFmM is detailed in Table 1.

**TABLE 1:** Chemical composition of IFmM

Compound	FW (g/mol)	Concn (mM)	mg/liter
Inorganic salts			
Calcium chloride	110.98	1.35	150.00
Magnesium sulfate anhydrous	120.00	0.83	100.00
Potassium chloride	75.00	5.33	400.00
Sodium chloride	58.44	102.67	6,000.00
Sodium phosphate dibasic anhydrous	142.00	5.63	800.00
Sodium bicarbonate	84.00	23.81	2,000.00
Vitamins			
d-Biotin	244.00	8.20E−04	0.20
Choline chloride	140.00	2.14E−02	3.00
d-Calcium pantothenate	477.00	5.24E−04	0.25
Folic acid	441.00	2.27E−03	1.00
*myo*-Inositol	180.16	1.94E−01	35.00
Niacinamide	122.00	8.20E−03	1.00
*p*-Amino benzoic acid	137.00	7.30E−03	1.00
Pyridoxine hydrochloride	206.00	4.85E−03	1.00
Riboflavin	376.00	5.32E−04	0.20
Thiamine hydrochloride	337.00	2.97E−03	1.00
Vitamin B_12_	1,355.00	3.69E−06	0.005
Amino acids			
Glycine	75.07	0.248	18.59
l-alanine	89.10	0.386	34.41
l-arginine	210.60	0.088	18.57
l-asparagine	132.12	0.062	8.13
l-aspartic acid	133.11	0.043	5.72
l-cysteine	157.62	0.034	5.36
l-glutamic acid	147.13	0.071	10.46
l-glutamine	146.15	0.773	112.99
l-histidine	209.63	0.117	24.57
l-isoleucine	131.18	0.069	9.05
l-leucine	131.18	0.163	21.42
l-lysine	182.65	0.175	31.91
l-methionine	149.21	0.033	4.95
l-phenylalanine	165.19	0.064	10.54
l-proline	115.13	0.050	5.75
l-serine	105.09	0.115	12.11
l-threonine	119.12	0.132	15.72
l-tryptophan	204.23	0.015	2.96
l-tyrosine	181.19	0.066	11.99
l-valine	117.15	0.249	29.19
Major carbon source			
Glucose	180.16	2.50	450

### C. burnetii replication potential in mammalian cell lines is cell type specific.

Previous studies have demonstrated that different C. burnetii strains exhibit unique replication potentials within a single cell type, including human alveolar macrophages (9). We sought to probe the amphotropic nature of C. burnetii by comparing intracellular replication of C. burnetii in the following four unique mammalian cell lines: the murine macrophage-like J774A.1, the human placental trophoblast-derived epithelial JEG-3, the human monocyte-derived THP-1, and the African green monkey kidney epithelial VERO cell lines. These cell lines represent three different cell types colonized by the bacterium, and J774A.1, THP-1, and VERO cells are widely used host cell models for analysis of C. burnetii pathogenesis. C. burnetii replication in placental trophoblast-derived cell lines has been described (33, 34).

**FIG 1:**
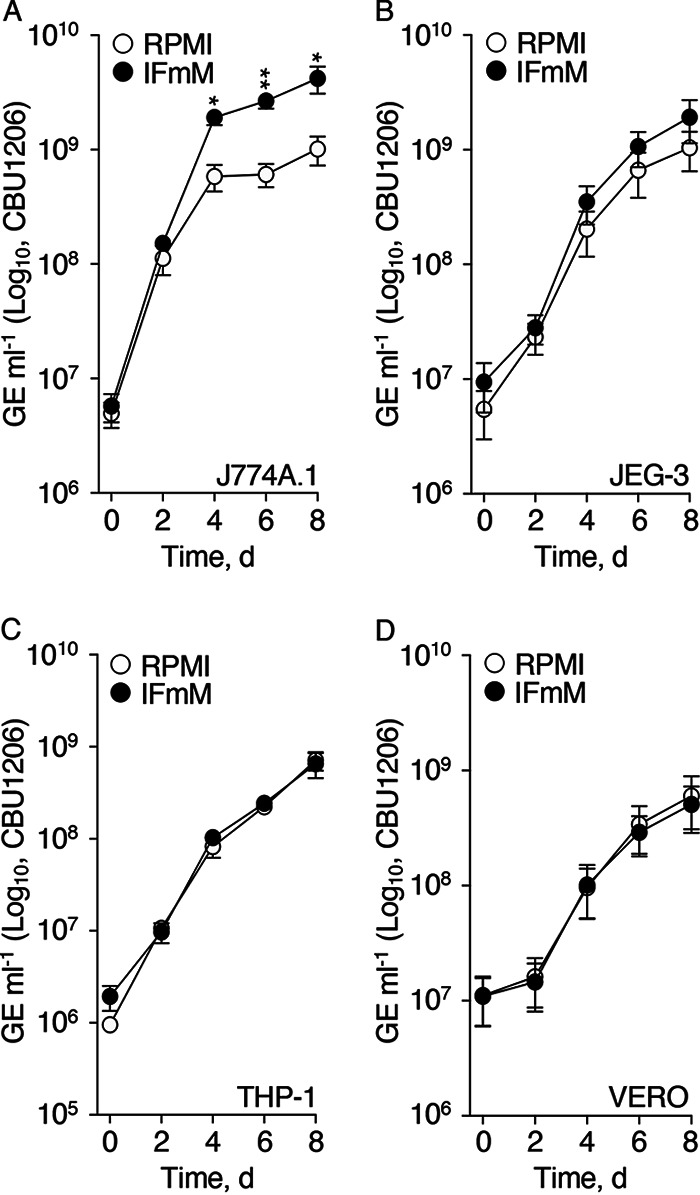
C. burnetii intracellular replication potential is dependent on host cell type and nutritional environment. C. burnetii intracellular replication was measured during infection of J774A.1 (A), JEG-3 (B), THP-1 (C), and VERO (D) cells cultured in complete RPMI or IFmM. Replication was determined via quantification of GE every 2 days for 8 days. Depicted data illustrate means ± SEM (*n* = 3 to 6). *, *P *< 0.05; **, *P *< 0.01 (unpaired Student's *t* test).

Host cells were infected with C. burnetii at a multiplicity of infection (MOI) (C. burnetii genome equivalents [GE] per host cell) of 5, and bacterial growth was determined by quantifying GE every 2 days for a total of 8 days. Because host nutrient availability is expected to influence intracellular replication of C. burnetii, replication potentials were compared between C. burnetii-infected host cells cultured in RPMI and our novel IFmM. C. burnetii replication in J774A.1 cells was indistinguishable between conditions for the first 2 days and then diversified to result in final bacterial burdens ∼4 times greater in cells maintained in IFmM (Fig. 1A). In contrast, C. burnetii replication in JEG-3, THP-1, and VERO cells was not significantly affected by the medium used (Fig. 1B to D). Of note, C. burnetii exhibited an extended 2-day lag phase during infection of VERO cells, regardless of culture medium (Fig. 1D). Overall, C. burnetii intracellular replication potential is dependent on the nutritional environment in a host cell type-specific manner.

### Medium glucose and amino acid concentrations directly influence C. burnetii intracellular yields.

Enhanced intracellular replication of C. burnetii in J774A.1 cells cultured in IFmM motivated assessment of host-mediated processes (e.g., pathogen uptake) and the influence of key nutrients on pathogen intracellular replication. Our results indicate that C. burnetii GE are comparable on day 0 and day 2 postinfection (p.i.) for naturally phagocytic cell lines (i.e., J774A.1 and THP-1), regardless of cell culture medium used (Fig. 1A and C). These data suggest that rates of phagocytosis are not affected by the cell culture media used in our analyses. Similar experiments performed using heat-killed bacteria confirmed that increases in bacterial loads during infection of host cells cultured in IFmM are a result of replication and not increased uptake of bacteria (data not shown).

**FIG 2:**
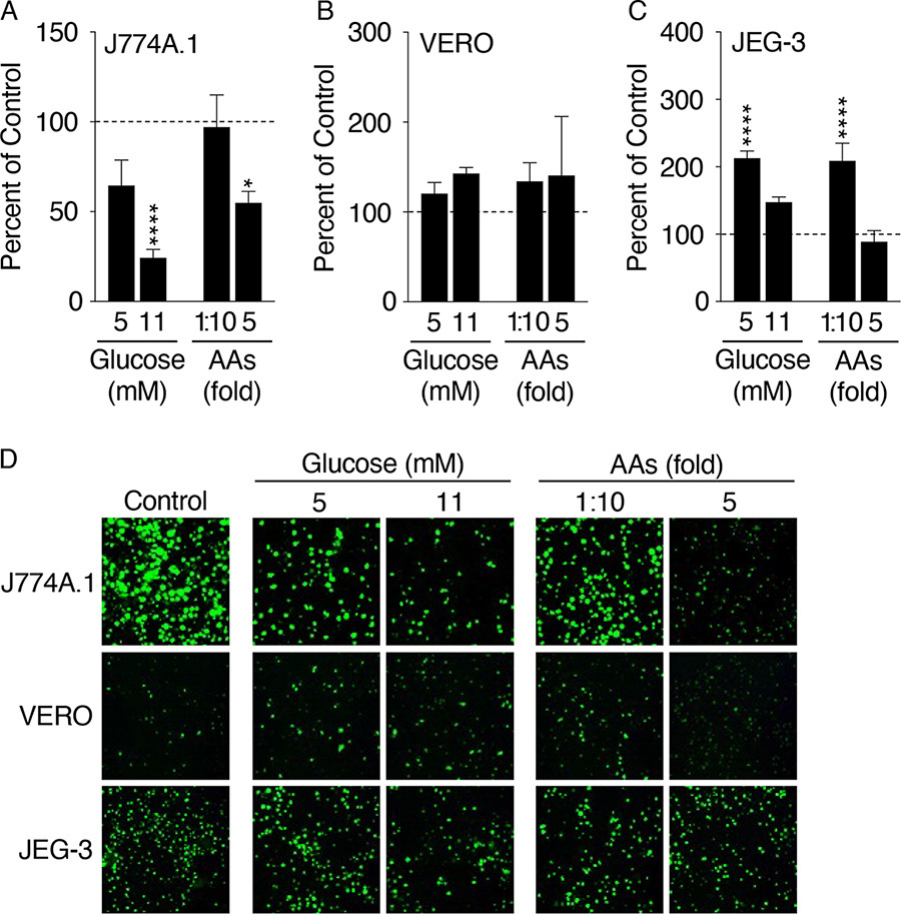
Medium glucose and amino acid levels directly influence C. burnetii intracellular replication in a cell type-dependent manner. To assess the influence of specific nutrients provided to the host on C. burnetii intracellular replication, J774A.1 (A), VERO (B), and JEG-3 (C) cells were infected with C. burnetii expressing GFP and cultured in modified IFmM with increasing amounts of glucose or altered concentrations of amino acids (AAs). C. burnetii yields were determined via enumeration of GE at 5 days p.i., and values presented as percent of control (complete IFmM, 100%, dashed line). Depicted data illustrate means ± SEM (*n* = 3 to 8). *, *P *< 0.05; ****, *P *< 0.0001 (one-way analysis of variance [ANOVA] with Dunnett’s posttest applied to panels A and C). (D) Representative fluorescence micrographs of GFP expressing C. burnetii 5 days p.i. under the conditions described for panels A to C.

Since uptake of bacteria was comparable between host cells cultured in RPMI or IFmM, focus was turned to potential effects of host nutrient access. IFmM differs from RPMI mainly in the concentrations of glucose and amino acids. Therefore, we evaluated the effect of altered medium glucose and amino acids on C. burnetii intracellular yields. J774A.1, VERO, and JEG-3 cells were infected with C. burnetii expressing green fluorescent protein (GFP) and cultured in IFmM, where (i) the glucose concentration was adjusted from 2.5 mM (control) to 5.5 or 11 mM (i.e., concentrations of glucose found in DMEM and MEM or RPMI, respectively; see Table S1), or (ii) amino acids were diluted 10-fold (i.e., amino acid starvation) or increased 5-fold (i.e., similar to the concentration of amino acids in RPMI; see Table S1). At 5 days p.i., cultures were processed for quantification of GE and imaged for qualitative assessment of CCV size. During infection of J774A.1 cells, increasing concentrations of glucose to 11 mM or amino acids 5-fold resulted in significantly reduced yields compared to those of complete IFmM (i.e., control, 100%) (Fig. 2A). When medium amino acids were diluted, however, C. burnetii yields were comparable to control conditions. Corresponding fluorescent micrographs showed smaller CCVs for conditions under which C. burnetii GE were reduced (Fig. 2D). In VERO cells, C. burnetii yields were comparable regardless of the medium alteration (Fig. 2B). Despite supporting similar growth yields, fluorescent foci in VERO cells incubated with elevated concentrations of amino acids revealed the presence of comparatively smaller CCVs (Fig. 2D). In JEG-3 cells, addition of 5 mM glucose or a 10-fold dilution in amino acids resulted in significantly increased C. burnetii yields (Fig. 2C). Increases in C. burnetii yields in JEG-3 cells correlated with the presence of larger CCVs based on fluorescent foci (Fig. 2D). These data suggest that medium glucose and/or amino acids directly influence C. burnetii intracellular replication in a cell type-dependent manner.

### Host cell types differ in intracellular pools of glucose and glutamate.

**FIG 3:**
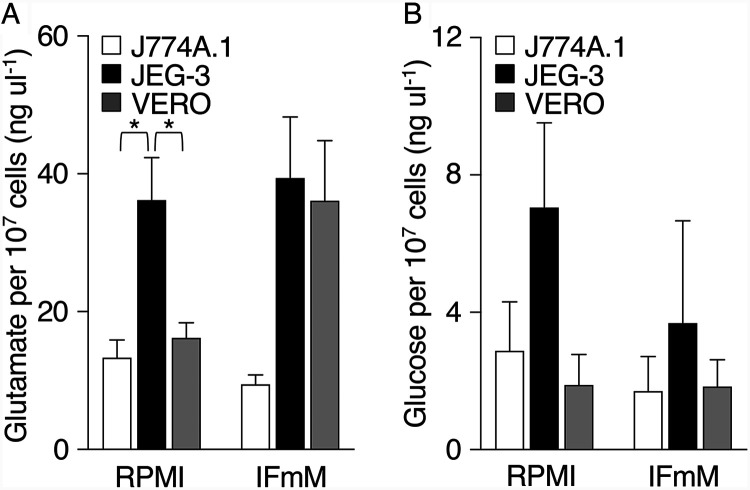
Intracellular pools of glutamate and glucose are host cell type specific. Intracellular concentrations of glutamate (A) and glucose (B) in J774A.1, JEG-3, and VERO cells maintained in RPMI or IFmM were quantified. Data are presented as mean intracellular concentration per 10^7^ cells as determined via direct cell counts. Depicted data illustrate the means ± SEM (*n* = 3 to 7). *P *< 0.05 (one-way ANOVA with Tukey’s posttest).

C. burnetii metabolic fitness is expected to be directly impacted by access to specific nutrients that are mainly acquired or produced by the host cell. Based on documented roles in C. burnetii physiology and data presented in Fig. 2, we measured the concentrations of glutamate (17, 35, 36) and glucose (24, 37) in J774A.1, JEG-3, and VERO cells maintained in RPMI versus IFmM. For J774A.1 and VERO cells cultured in RPMI, the mean concentrations (±standard error of the mean [SEM]) of intracellular glutamate were similar (i.e., glutamate_J774A.1_ = 13.32 ± 2.55 ng μl^−1^; glutamate_VERO_ = 16.17 ± 2.23 ng μl^−1^) and significantly lower than the concentration measured in JEG-3 cells (36.19 ± 6.15 ng μl^−1^) (Fig. 3A). In comparison, when cultured in IFmM, intracellular glutamate concentrations in J774A.1 cells (9.46 ± 1.34 ng μl^−1^) remained lower than those measured for JEG-3 (39.38 ± 8.87 ng μl^−1^) and VERO cells (36.09 ± 8.72 ng μl^−1^). Moreover, VERO cells cultured in IFmM contained an ∼2-fold higher concentration of glutamate than cells cultured in RPMI (*P = *0.0513) (Fig. 3A). Measurements for intracellular glucose pools were highly variable between experiments for all cell lines, with <10 ng μl^−1^ glucose measured regardless of cell type or culture medium (Fig. 3B). Overall, intracellular pools of glutamate and glucose—major carbon sources for C. burnetii—are host-cell specific and can change based on medium nutrient composition.

### Gluconeogenic capacity is necessary for optimal C. burnetii amphotropic fitness.

**FIG 4:**
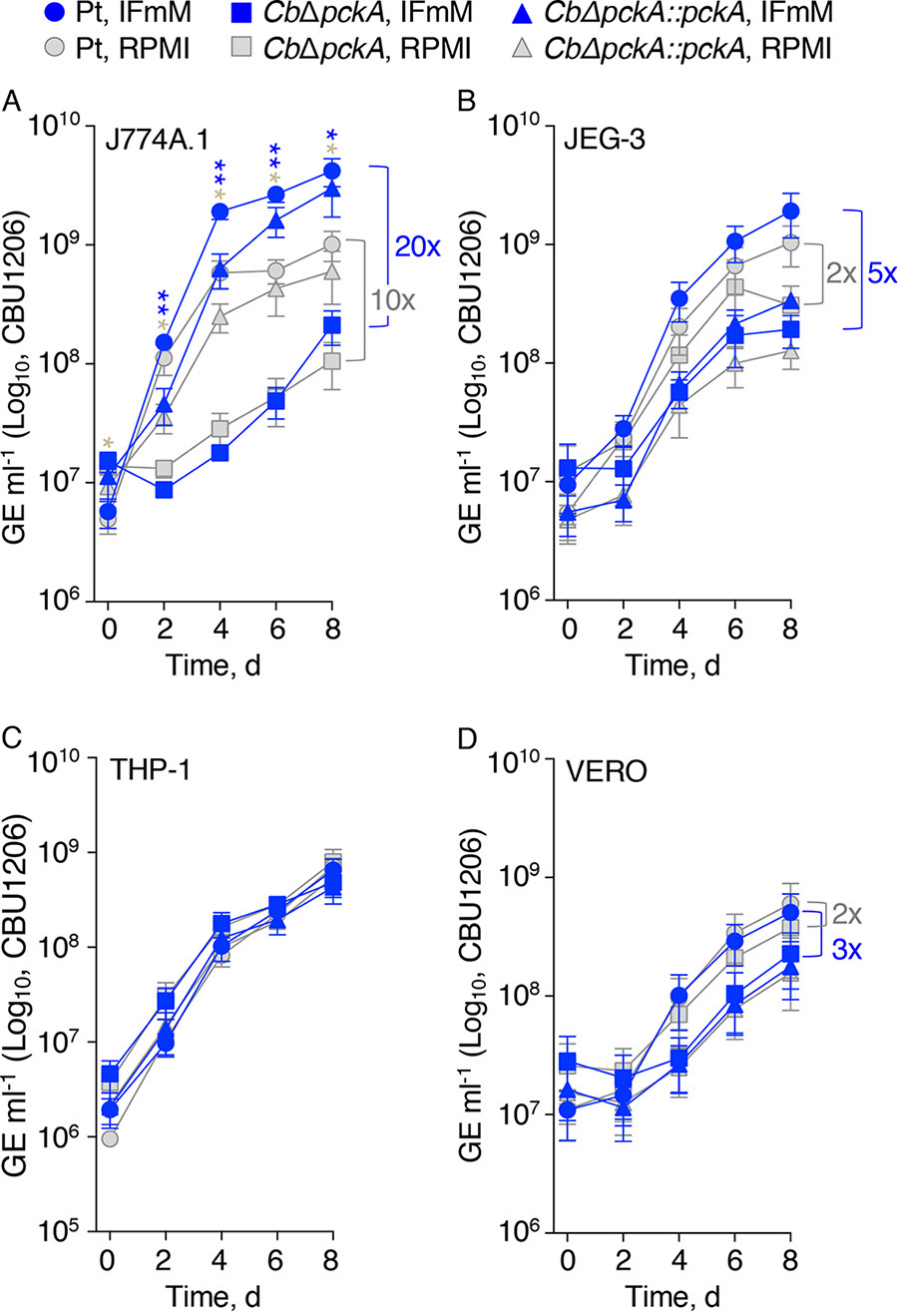
Gluconeogenic capacity is necessary for C. burnetii amphotropic fitness. Intracellular growth of the Pt, *Cb*Δ*pckA*, and *Cb*Δ*pckA*::*pckA* strains were compared during infection of J774A.1 (A), JEG-3 (B), THP-1 (C), and VERO (D) cells cultured in IFmM (blue) or RPMI (gray). Replication was determined via quantification of GE every 2 days for 8 days. Depicted data illustrate means ± SEM (*n* = 3 to 6). Asterisks in blue and gray indicate differences between the Pt and *Cb*Δ*pckA* strains during infection of host cells cultured in IFmM or RPMI, respectively. *, *P *< 0.05; **, *P *< 0.01 (unpaired Student's *t* test). Brackets indicate the change in final (i.e., day 8) yields between the Pt and *Cb*Δ*pckA* strains.

Deletion of the gene *pckA*, encoding phosphoenolpyruvate carboxykinase, prevents gluconeogenic activity in C. burnetii. Previously, the *pckA* deletion mutant *Cb*Δ*pckA* was determined to have a moderate (glucose-dependent) growth defect in VERO cells cultured in RPMI (24). To test whether abolishment of the bacterium’s gluconeogenic capacity affects its replication potential in biologically diverse cell lines (i.e., amphotropic fitness), intracellular replication of parental C. burnetii (Pt; control) was compared to that of the *Cb*Δ*pckA* mutant and its functional complement *Cb*Δ*pckA*::*pckA* in J774A.1, JEG-3, THP-1, and VERO cells. In J774A.1 cells maintained in IFmM, the *Cb*Δ*pckA* mutant had a severe (∼1.5 to 2 log) growth defect from 2 to 8 days p.i. compared to the Pt strain (Fig. 4A, blue). In JEG-3 cells maintained in IFmM, the *Cb*Δ*pckA* mutant tended to exhibit reduced growth from 4 to 8 days p.i. compared to the Pt strain (Fig. 4B, blue). Unlike in J774A.1 cells, complementation of the *Cb*Δ*pckA* mutant with *pckA* (*Cb*Δ*pckA*::*pckA*) did not restore growth within the JEG-3 cell line (Fig. 4B), demonstrating that complete recovery by the *pckA*-complemented strain is dependent on the host cell type. In THP-1 cells, no difference in overall growth was observed for either the *Cb*Δ*pckA* or *Cb*Δ*pckA*::*pckA* strain compared to that of the Pt strain in IFmM (Fig. 4C, blue). As previously observed (24), the *Cb*Δ*pckA* mutant had a moderate growth defect in VERO cells maintained in RPMI (Fig. 4D, gray), and this defect was observed also in IFmM (Fig. 4D, blue). Differences in final yields between the Pt and *Cb*Δ*pckA* strains were generally augmented when host cells were cultured in IFmM compared to RPMI as follows: J774A.1 (20-fold in IFmM versus 10-fold in RPMI; *P = *0.0623), JEG-3 (5-fold in IFmM versus 2-fold in RPMI; *P = *0.0352), and VERO (3-fold in IFmM versus 2-fold in RPMI; *P = *0.3369). The use of IFmM did not affect replication of either the Pt or *Cb*Δ*pckA* strain in THP-1 cells (Fig. 4, gray versus blue). Overall, these data demonstrate that gluconeogenic capacity enhances C. burnetii amphotropic fitness and that use of IFmM exacerbates the gluconeogenic defect of the *Cb*Δ*pckA* mutant in a host cell type-dependent manner.

### The Drosophila melanogaster animal model reveals a role for metabolic plasticity in C. burnetii virulence.

**FIG 5:**
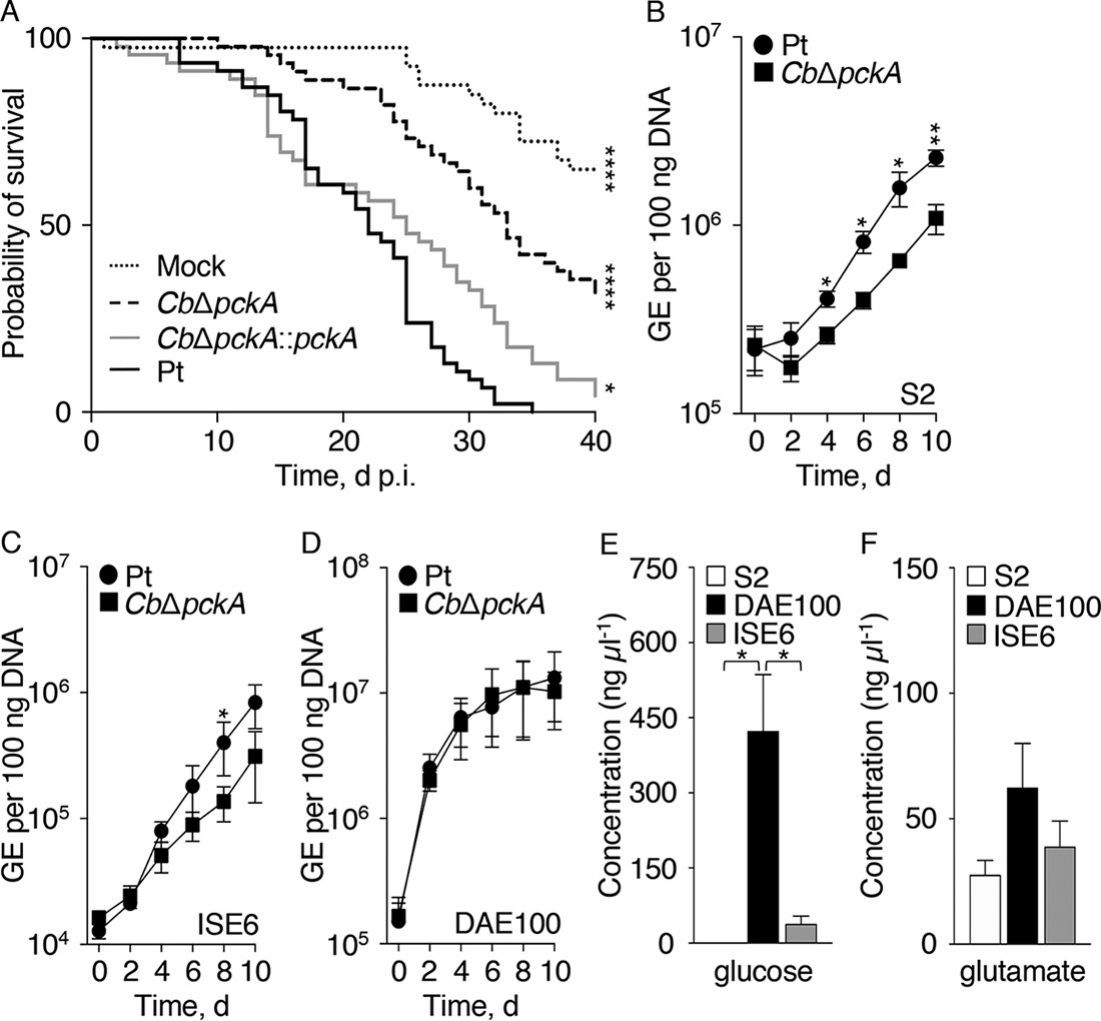
Metabolic plasticity enhances C. burnetii virulence. (A) D. melanogaster survival was assessed daily for 40 days following mock challenge or infection with the Pt, *Cb*Δ*pckA*, and *Cb*Δ*pckA*::*pckA* strains. Depicted data illustrate a single experiment (*n* = 50 flies) and is representative of 2 independent experiments. *, *P *< 0.05; ****, *P *< 0.0001 (Mantel-Cox compared to control). The ability of the *Cb*Δ*pckA* strain to infect D. melanogaster S2 (B), Ixodes scapularis (ISE6) (C), and Dermacentor andersoni (DAE100) (D) cells was compared to the Pt strain every 2 days for 10 days. Depicted data illustrate means ± SEM (*n* = 3 to 4). *, *P *< 0.05; **, *P *< 0.01 (unpaired Student's *t* test). Quantification of intracellular concentrations of glucose (E) and glutamate (F) in homogenates of S2, DAE100, and ISE6 cells. Depicted data illustrate means ± SEM (*n* = 3 to 5). *, *P *< 0.05 (one-way ANOVA with Tukey’s posttest).

The growth defect displayed by the *Cb*Δ*pckA* mutant in certain mammalian cell lines suggested that metabolic plasticity enhances C. burnetii amphotropic fitness. To assess whether C. burnetii metabolic plasticity can similarly impact pathogen virulence, the established Drosophila melanogaster animal model (38) was employed. D. melanogaster survival was quantified every day for 40 days, following challenge with the Pt, *Cb*Δ*pckA*, and *Cb*Δ*pckA*::*pckA* strains, or phosphate-buffered saline (PBS) (mock). Both the Pt and *Cb*Δ*pckA*::*pckA* strains were equally virulent with ≤4% of the infected flies surviving relative to mock infected flies at 40 days p.i. (Fig. 5A). In contrast, roughly 30% of flies infected with the *Cb*Δ*pckA* mutant survived, a significantly higher proportion compared to the level observed following infection with the Pt or *Cb*Δ*pckA*::*pckA* strain (Fig. 5A). To determine whether morbidity of infected flies correlated with an increase in bacterial burdens, C. burnetii GE were quantified from flies at 15 and 30 days p.i. C. burnetii burdens were comparable for flies infected with the Pt and *Cb*Δ*pckA* strains, regardless of time point (see Fig. S1 in the supplemental material). Therefore, gluconeogenic capacity is required for optimal virulence via a mechanism that does not depend on overall fly colonization.

The ability of the *Cb*Δ*pckA* mutant to replicate in flies and reach overall burdens similar to the Pt strain, yet exhibit a defect in virulence, suggested that the mutant was incapable of colonizing specific cells critical for fly viability. Because D. melanogaster-derived S2 hemocytes (39) are susceptible to C. burnetii (38), we assessed intracellular replication of the *Cb*Δ*pckA* mutant in this professional macrophage-like cell type as well as in the Dermacentor andersoni DAE100 and Ixodes scapularis ISE6 tick-derived cell lines. Unlike S2 hemocytes, DAE100 and ISE6 cells represent arthropod-derived cell lines of embryonic origin shown to differ in phagocytic activity (40). *Cb*Δ*pckA* mutant replication potentials were compared to the Pt strain every 2 days for 10 days. While bacterial loads immediately after infection (i.e., day 0) were equivalent, the *Cb*Δ*pckA* strain replicated less effectively than the Pt strain in S2 and ISE6 cells, resulting in significantly reduced bacterial loads at one or more time point from day 4 to 10 (Fig. 5B and C). In comparison, growth of the *Cb*Δ*pckA* mutant was not significantly different from that of the Pt strain in DAE100 cells (Fig. 5D). Although factors other than access to glucose could affect replication of the *Cb*Δ*pckA* mutant, both S2 and ISE6 cells contained significantly lower concentrations of glucose (i.e., glucose_S2_ = 0.22 ± 0.04 ng μl^−1^; glucose_ISE6_ = 38.35 ± 15.68 ng μl^−1^) compared to that measured for DAE100 cells (423.57 ± 112.55 ng μl^−1^) (Fig. 5E). In comparison, ∼20 to 80 ng μl^−1^ glutamate was measured for each of the arthropod cell lines tested, but differences between cell lines were not statistically significant (Fig. 5F). These data are consistent with significance of gluconeogenic capacity for C. burnetii intracellular replication within specific arthropod cell types.

## DISCUSSION

C. burnetii is a zoonotic pathogen with an extensive host range, including humans, domestic animals, wildlife, and arthropods. While determinants for species-specific colonization are poorly defined, carriage of the plasmid QpH1 has been linked to C. burnetii colonization of murine macrophages (41). Upon infection, C. burnetii colonizes numerous tissues and cell types consistent with a requirement of the pathogen to adapt to nutritionally diverse environments. Interstitial fluid (IF)—a conduit for nutrients that connects blood plasma with the cellular constituents of tissues—is a physiologically relevant nutritional environment supporting most *Coxiella*-mammalian host interactions. Accordingly, we designed a physiological medium, IFmM, which better reflects the nutrient composition of IF to test C. burnetii replication phenotypes in different cell types (Fig. 1). The significance of metabolic plasticity in C. burnetii amphotropism is largely unexplored. In this study, we sought to identify whether a connection exists between the ability of C. burnetii to replicate in different cell types and plasticity in central metabolism. To this end, C. burnetii intracellular replication in seven distinct cell lines (i.e., four mammalian- and three arthropod-derived) were compared between a metabolically competent parental strain and the gluconeogenesis-deficient *Cb*Δ*pckA* strain. As expected, both the parental strain and mutant exhibited host cell-dependent intracellular replication phenotypes. Moreover, different intracellular levels of glycolytic (i.e., glucose) and gluconeogenic (i.e., glutamate) substrates in different cell types are consistent with a requirement for metabolic plasticity to facilitate C. burnetii amphotropism.

Because nutrient media can shape the physiology of cultured cells (42), the use of so-called “physiological media” (i.e., media that better reflect physiological conditions) have seen a recent spike in interest (43). Similarly, as observed with Salmonella enterica, medium composition (i.e., high concentration of glucose) can influence pathogen intracellular replication and mask the metabolic state of the bacterium during infection (44). Unlike highly specialized physiological media designed for culture and analysis of specific cell types, such as neurons (45), or that mimic blood serum (43, 46), IFmM was designed based on the chemical composition of IF. Similar concepts have been used to model airway infections associated with Bordetella bronchiseptica (47) and exacerbation of cystic fibrosis (48). Our model, with medium replacement every 2 d to better mimic natural steady state conditions in nutrient availability, resulted in growth kinetics for C. burnetii akin to those observed using commercial RPMI for some but not all host cell lines (Fig. 1). Importantly, enhanced C. burnetii intracellular replication in certain host cells cultured in IFmM was not a consequence of altered phagocytosis (data not shown). Beyond applicability in the analysis of C. burnetii physiology and virulence, IFmM may prove useful in the study of other host and host-pathogen systems for which IF serves as the natural conduit of nutrients. For instance, the obligate intracellular bacterium Chlamydia trachomatis, which infects mucosal epithelial cells and is dependent on scavenging a wide range of nutrients from the host (49), replicated similarly in host cells cultured in IFmM compared to those cultured in RPMI (see Fig. S2 in the supplemental material).

Recent analysis of C. burnetii carbon metabolism in either cell culture or under axenic conditions have determined that the bacterium is not dependent on exogenous glucose for replication (24, 50) but employs a bipartite-type carbon metabolism that uses both glycolytic and gluconeogenic substrates (18, 24, 51). In comparison, C. burnetii was shown to favor glucose for anabolism versus catabolism of fatty acids for energy metabolism within the spleen of mice (52). In this work, we identify a link between specific host cell types and metabolic requirements of C. burnetii (e.g., Fig. 1 and 5). We provide evidence that small alterations to individual metabolic substrates (e.g., glucose or amino acids) supplied to the host directly influence C. burnetii replication potentials and that these responses are host cell-type specific (Fig. 2A and C). Moreover, we show that medium nutrients influence CCV size (Fig. 2D), consistent with altered C. burnetii activity.

Metabolic plasticity in central carbon metabolism is critical for several pathogens to successfully establish infection. For instance, Mycobacterium tuberculosis fails to establish and maintain infection in the absence of gluconeogenic capacity even though the pathogen can utilize various carbon sources (53). Using a *pckA* deletion mutant of C. burnetii, we show that gluconeogenesis is critical for optimal replication within the physiologically distinct J774A.1, JEG-3, and VERO cells (Fig. 4). Of note, the effect of *trans*-complementating the *CbΔpckA mutant* was variable and the *Cb*Δ*pckA*::*pckA* strain exhibited cell line-dependent growth phenotypes. Therefore, constitutive expression of *pckA* can negatively impact C. burnetii intracellular replication, consistent with a need for C. burnetii to negatively regulate *pckA* expression and/or activity for optimal fitness.

The C. burnetii reference strain Nine Mile I was isolated from an arthropod host, specifically from a tick (54). In recent years, arthropod models have been established for C. burnetii, including the fruit fly D. melanogaster (38) and greater wax moth Galleria mellonella (55). Within these organisms, C. burnetii colonizes several cells and tissues including hemocytes (38, 55, 56), the midgut, salivary glands, and ovaries (57, 58). This is consistent with the natural biology of C. burnetii having evolved to infect and replicate within physiologically and evolutionarily distinct cell types. Using the *Drosophila* model, we highlight that the *Cb*Δ*pckA* strain exhibits a virulence defect (Fig. 5A) despite being able to colonize flies (see Fig. S1 in the supplemental material). This defect in virulence correlated with a moderate replication defect observed upon infecting D. melanogaster S2 cells with the *CbΔpckA* mutant (Fig. 5B). The lack of a replication defect, however, for the *Cb*Δ*pckA* strain infecting DAE100 cells highlights that C. burnetii infection phenotypes are host-cell specific also in arthropod-derived cell lines. Francisella novicida has been shown to reach higher bacterial burdens and cause increased cytotoxicity in DAE100 cells compared to that in ISE6 cells (40).

In conclusion, we have demonstrated a requirement of C. burnetii core-metabolic plasticity for optimal replication and virulence in physiologically and evolutionary diverse cell types (i.e., amphotropic fitness). We also show that the use of an IF-based physiological medium facilitates resolution of C. burnetii intracellular replication phenotypes. Overall, our results indicate that consideration of C. burnetii amphotropism is critical when attempting to resolve virulence mechanisms in this pathogen.

## MATERIALS AND METHODS

### Bacterial strains.

C. burnetii Nine Mile phase II (NMII) (RSA 439, clone 4) was propagated in ACCM-2 and prepared for long-term storage as described (59, 60). Culture inocula were normalized to GE (60).

### Composition and preparation of interstitial fluid-modeled medium.

The chemical composition of IFmM is shown in Table 1. Complete IFmM was prepared by mixing the base medium consisting of inorganic salts (pH 7.2), RPMI 1640 vitamins (Thermo Fisher Scientific, Waltham, MA), glucose, amino acids, and 5% (vol/vol) heat-inactivated serum complex (hiFetalPlex; Gemini Bio Products, West Sacramento, CA). Final pH was adjusted to 7.2 to 7.4 and the medium was filter sterilized (0.22 μm) and stored at 4°C.

### Cell culture and infection.

Mouse macrophage-like J774A.1 (TIB-67, ATCC), African green monkey kidney epithelial VERO (CCL-81, ATCC), and human placental trophoblast-derived epithelial JEG-3 (HTB-36, ATCC) cells were maintained in complete RPMI 1640 medium (i.e., RPMI 1640 without l-glutamine [Corning cellgro; Corning Inc., Corning, NY]) supplemented with GlutaMAX (Gibco BioSciences, Dublin, Ireland) and 5% (vol/vol) hiFetalPlex at 37°C and 5% CO_2_. Human monocyte-derived THP-1 (TIB-202; ATCC) cells were maintained in RPMI 1640 medium containing 2 mM l-glutamine (Gibco BioSciences, Dublin, Ireland), 10 mM HEPES (HyClone; GE Life Sciences, Pittsburgh, PA), 1.0 mM sodium pyruvate and 10% hiFetalPlex at 37°C and 5% CO_2_. Unless otherwise stated, host cells were seeded in 6-well or 12-well plates at a density of 2 × 10^5^ cells per 3 or 1.5 ml, respectively, and maintained in complete RPMI 1640 at 37°C and 5% CO_2_ for 24 h. Subsequently, host cells were infected at an MOI of 5 in 1 ml of plain RPMI 1640. Infections were facilitated by centrifugation at 400 × *g* for 30 min at room temperature (RT). Following infection, host cells were washed 3 times with IFmM inorganic salts solution (pH 7.2) to remove unattached and noninternalized bacteria before complete IFmM or RPMI 1640 infection medium (i.e., RPMI 1640 without l-glutamine supplemented with GlutaMAX and 2% [vol/vol] hiFetalPlex) was added to each well. Media were replenished every 48 h for the duration of the 8-day infection cycle. At the time of sampling, host cells from duplicate wells were detached via gentle scraping or use of trypsin and transferred to 1.5-ml tubes for processing and quantification of bacterial GE. Samples for the zero hour time point were collected ∼2 h postinfection.

*Drosophila* Schneider 2 (S2) cells (Thermo Fisher Scientific, Waltham, MA) were maintained in Schneider’s insect medium (Sigma-Aldrich, St. Louis, MO) containing 10% (vol/vol) hiFetalPlex at 28°C. Dermacentor andersoni DAE100 and Ixodes scapularis ISE6 cells were maintained in complete L15B medium (61) at 34°C. For infections, S2 cells were seeded in 12-well plates at a density of ∼1 × 10^6^ cells per 1 ml, while DAE100 and ISE6 cells were seeded in 12-well plates by diluting confluent cell cultures 1:2 or 1:5, respectively. S2 cells were infected using 0.5 ml of plain Schneider’s insect medium containing 1 × 10^7^ GE ml^−1^ of C. burnetii for 24 h at 28°C, and tick cells were infected using 1 ml of complete L15B containing 1 × 10^7^ GE ml^−1^ of C. burnetii for 24 h at 34°C. Following infection, inocula were removed, and 1 ml of Schneider’s insect medium containing 2% (vol/vol) hiFetalPlex or complete L15B was added to each well for the S2 and DAE100/ISE6 cells, respectively. Arthropod infections were allowed to progress for 10 days, with samples taken every 48 h. At time of sampling, cultures from duplicate wells were transferred to 1.5-ml tubes and host cells pelleted via centrifugation for 10 min at 9,000 × *g*. Supernatants were removed and pellets stored at −20°C until GE quantification.

### Quantification of intracellular glucose and glutamate pools.

Intracellular glucose and glutamate pools were quantified using commercially available assay kits (Sigma-Aldrich, St. Louis, MO) according to manufacturer’s instructions. For J774A.1, JEG-3, VERO, and S2 cell lines, 2 to 4 confluent T-75 flasks were used for the analyses. JEG-3 and VERO cultures were harvested via trypsinization, while J774A.1 cultures were harvested by gentle scraping. For the DAE100 and ISE6 cell lines, 2 or 3 confluent T-25 flasks were harvested via repeated pipetting using a serological pipette. Cells were washed twice with PBS (pH 7.2) before cell numbers were determined via direct cell counts for normalization purposes. Finally, cells were pelleted and resuspended in 0.25 ml of PBS containing 0.1% (wt/vol) SDS, heated for 10 min at 95 to 100°C to inactivate host enzymes, and then subsequently centrifuged for 10 min at 10,000 × *g* (RT). The resulting supernatants were assayed directly.

### C. burnetii infections of Drosophila melanogaster.

Male D. melanogaster w^1118^ flies were infected and survival monitored as described (38). Briefly, adult flies were infected with bacteria at 1 × 10^5^ GE via direct injection into the fly thorax using a nanoliter injector (Drummond Scientific, Broomall, PA). Following injection, fly mortality was determined every 24 h over 40 days with flies collected at 15 and 30 days p.i. to determine relative bacterial loads by measuring GE.

### Measurement of genome equivalents.

Quantification of bacteria by GE was performed as described (60). Briefly, 1 ml of C. burnetii NMII cultures was added to a 1.5-ml screw-cap tube containing 0.1-mm zirconia beads (Bio Spec Products, Bartlesville, OK) and subjected to mechanical lysis (FastPrep-24; MP Biomedicals, Irvine, CA) via three, 30-s pulses at 5.0 m s^−1^. Samples were serially diluted and GE quantified via detection of the C. burnetii gene CBU1206 (62) using a CFX96 real-time PCR detection system (Bio-Rad Laboratories, Hercules, CA) and the iTaq Universal SYBR green Supermix (Bio-Rad Laboratories, Hercules, CA). C. burnetii GE was extrapolated from a standard curve prepared using recombinant CBU1206 (62).

To release bacterial DNA from flies, 3 flies per condition were added to a single Lysis Matrix H tube (MP Biomedicals, Santa Ana, CA) containing 0.3 ml of molecular grade water. Tubes were subjected to an initial round of mechanical homogenization at 6.0 m s^−1^ for 20 s and then centrifuged for 10 min at 100 × *g* to remove host debris. The supernatant was then transferred to a fresh tube containing 0.1-mm zirconia beads and mechanically homogenized via three, 30-s pulses at 5 m s^−1^. Any remaining debris was pelleted via centrifugation for 1 to 2 min at 10,000 × *g*. The resulting supernatant was used as template in quantitative PCRs.

Total DNA was extracted from C. burnetii-infected S2, ISE6, and DAE100 cells using the Nucleospin tissue kit (Macherey-Nagel, Düren, Germany). Briefly, cell pellets were resuspended in 0.18 ml T1 buffer and supplemented with 0.025 ml of proteinase K. Cells suspensions were incubated overnight at 56°C, and subsequent DNA extraction followed manufacturer’s instructions. Total DNA was eluted in 0.1 ml of 70°C molecular grade water. For GE quantification, 10 to 100 ng total DNA was used as template.

### Statistical analysis.

Statistical analysis was done using Prism software (GraphPad, CA). Except for C. burnetii infection of D. melanogaster, depicted data show the results from at least 3 independent experiments (*n*).
