# Supplementary material for: Metabolic Plasticity Aids Amphotropism of Coxiella burnetii
Source: Infect Immun. 2021 Nov 16;89(12):e00135-21. doi: 10.1128/IAI.00135-21 (PMC8594591; doi:10.1128/IAI.00135-21)
Supplement: Supplemental file 1 — Supplemental material. Download iai.00135-21-s0001.pdf, PDF file, 0.2 MB [file iai.00135-21-s0001.pdf]

Metabolic Plasticity Aids Amphotropism of *Coxiella burnetii*

SUPPLEMENTAL MATERIAL

Savannah E. Sanchez[a,b] , Alan G. Goodman[a,b], and Anders Omsland[a#]

[a]Paul G. Allen School for Global Health, and [b]School of Molecular Biosciences, College of Veterinary Medicine, Washington State University, Pullman, WA 99164.

[#]Address correspondence to Anders Omsland: anders.omsland@wsu.edu

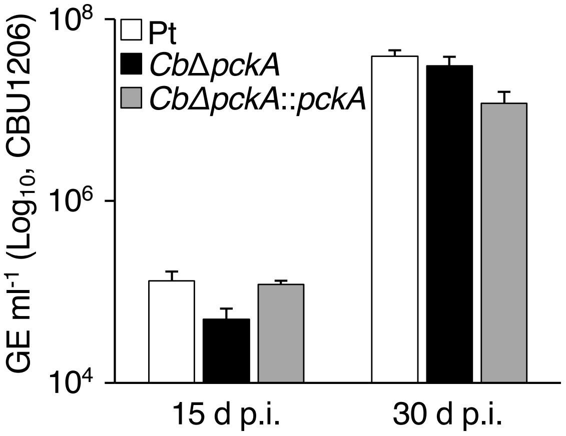

Figure S1. *C. burnetii* loads in *D. melanogaster* during infection. Infected flies were used for GE analysis of the Pt strain, *CbΔpckA,* and *CbΔpckA::pckA* at 15 and 30 d p.i.. Depicted data illustrate the mean ± SEM (n = 3).

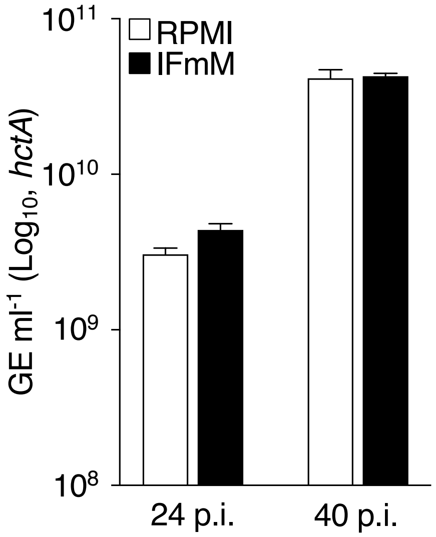

Figure S2. Replication of *Chlamydia trachomatis* in HeLa cells maintained in IFmM. *C. trachomatis* L2 intracellular replication was measured during infection of HeLa cells cultured in complete IFmM and compared to that observed in HeLa cells cultured in RPMI. Replication was determined via quantification of GE at 24 and 40 h p.i.. Depicted data illustrate the mean ± SEM (n = 3). The gene target for qPCR was *C. trachomatis hctA*.

Table S1. Comparison of IFmM and commercially available tissue culture media.

		IFmM		RPMI 1640	DMEM	MEM
Inorganic salts	FW (g/mol)	Concentration (mM)	mg/L	mg/L	mg/L	mg/L
Calcium chloride	110.98	1.35	150.00	-	200.00	200.00
Calcium nitrate	164.09	-	-	100.00	-	-
Ferric nitrate nonahydrate	403.99	-	-	-	0.10	-
Magnesium sulfate anhydrous	120.00	0.83	100.00	48.84	97.67	97.67
Potassium chloride	75.00	5.33	400.00	400.00	400.00	400.00
Sodium chloride	58.44	102.67	6000.00	6000.00	6400.00	6800.00
Sodium phosphate monobasic anhydrous	119.98	-	-	-	109.00	140.00
Sodium phosphate dibasic anhydrous	142.00	5.63	800.00	800.00	-	-
Sodium bicarbonate	84.00	23.81	2000.00	2000.00	3700.00	2200.00
Vitamins	FW (g/mol)	Concentration (mM)	mg/L	mg/L	mg/L	mg/L
D-Biotin	244.00	8.20E-04	0.20	0.20	-	-
Choline chloride	140.00	2.14E-02	3.00	3.00	4.00	1.00
D-Calcium pantothenate	477.00	5.24E-04	0.25	0.25	4.00	1.00
Folic acid	441.00	2.27E-03	1.00	1.00	4.00	1.00
myo-Inositol	180.16	1.94E-01	35.00	35.00	7.20	2.00
Niacinamide	122.00	8.20E-03	1.00	1.00	4.00	1.00
p-Amino benzoic acid	137.00	7.30E-03	1.00	1.00	-	-
Pyridoxal hydrochloride	203.62	-	-	-	4.00	1.00
Pyridoxine hydrochloride	206.00	4.85E-03	1.00	1.00	-	-
Riboflavin	376.00	5.32E-04	0.20	0.20	0.40	0.10
Thiamine hydrochloride	337.00	2.97E-03	1.00	1.00	4.00	1.00
Vitamin B12	1355.00	3.69E-06	0.005	0.005	-	-
Amino acids	FW (g/mol)	Concentration (mM)	mg/L	mg/L	mg/L	mg/L
Glycine	75.07	0.248	18.59	10.00	30.00	-
L-alanine	89.10	0.386	34.41	-	-	-
L-arginine	210.60	0.088	18.57	200.00	84.00	126.00
L-asparagine	132.12	0.062	8.13	50.00	-	-
L-aspartic acid	133.11	0.043	5.72	20.00	-	-
L-cysteine	157.62	0.034	5.36	-	-	-
L-cystine	240.00	-	-	65.20	62.60	31.00
L-glutamic acid	147.13	0.071	10.46	20.00	-	-

L-glutamine	146.15	0.773	112.99	300.00	584.00	292.00
L-histidine	209.63	0.117	24.57	15.00	42.00	42.00
L-hydroxyproline	131.00	-	-	20.00	-	-
L-isoleucine	131.18	0.069	9.05	50.00	105.00	52.00
L-leucine	131.18	0.163	21.42	50.00	105.00	52.00
L-lysine	182.65	0.175	31.91	40.00	146.00	73.00
L-methionine	149.21	0.033	4.95	15.00	30.00	15.00
L-phenylalanine	165.19	0.064	10.54	15.00	66.00	32.00
L-proline	115.13	0.050	5.75	20.00	-	-
L-serine	105.09	0.115	12.11	30.00	42.00	-
L-threonine	119.12	0.132	15.72	20.00	95.00	48.00
L-tryptophan	204.23	0.015	2.96	5.00	16.00	10.00
L-tyrosine	181.19	0.066	11.99	28.83	103.79	52.00
L-valine	117.15	0.249	29.19	20.00	94.00	46.00
Additional factors	FW (g/mol)	Concentration (mM)	mg/L	mg/L	mg/L	mg/L
Glucose	180.16	2.50	450	2000.00	1000.00	1000.00
Glutathione	307.32	-	-	1.00		-
Sodium pyruvate	110.00		-	-	110.00	-

Table S2. Comparison of concentrations of amino acids and ions in mammalian interstitial fluid to the chemical composition of IFmM.

	Final concentration (μM)			Final concentration (mM)	
Amino acids	Reported	IFmM	Ions	Reported	IFmM[g]
Alanine	248.0[a], 333.0[b], 386.2[c]	386.2	Na^+	155.1[d], 134.6[e]	137.75
Arginine	88.2[c]	88.2	K^+	5.2[d], 3.17[e]	5.33
Asparagine	52.0[a], 56.0[b], 61.5[c]	61.5	Ca^+	1.47[d], 1.55[e]	1.35
Aspartic acid	62.0[a], 43.0[b]	43.0	Mg^{+2}	0.51[e]	0.83
Cysteine	34.0[a], 34.0[b]	34.0	Cl^-	107.1[d]	110.70
Glutamic acid	43.0[a], 66.0[b], 71.1[c]	71.1	PO_4^{-3}	0.61[e]	5.63
Glutamine	529.0[a], 672.0[b], 773.1[c]	773.1	CO_2/HCO_3^-	23.9[e]	23.81
Glycine	565.0[a], 400.0[b], 247.6[c]	247.6			
Histidine	117.2[c]	117.2			
Isoleucine	65.0[a], 70.0[b], 69.0[c]	69.0			
Leucine	149.0[a], 165.0[b], 163.3[c]	163.3			
Lysine	174.7[c]	174.7			
Methionine	18.0[a], 14.0[b], 33.2[c]	33.2			
Phenylalanine	58.0[a], 72.0[b], 63.8[c]	63.9			
Proline	---	50.0			
Serine	195.0[a], 125.0[b], 115.2[c]	115.2			
Threonine	163.0[a], 185.0[b], 132.0[c]	132.0			
Tryptophan	14.5[c]	14.5			
Tyrosine	54.0[a], 62.0[b], 66.2[c]	66.2			
Valine	222.0[a], 257.0[b], 249.2[c]	249.2			

[a]Table 2, interstitial concentrations from adipose tissue (1).

[b]Table 2, interstitial concentrations from muscle tissue (1).

[c]Table 1, Period 3 (151-225 min) dialysate concentrations (2).

[d]Table 4, tissue fluid free concentrations (3).

[e]Table 2, interstitial fluid concentrations (4).

[g]Final ion composition of IFmM does not include ions contributed by amino acid salts.

REFERENCES

1. Maggs DG, Jacob R, Rife F, Lange R, Leone P, During MJ, Tamborlane WV, Sherwin RS. 1995. Interstitial fluid concentrations of glycerol, glucose, and amino acids in human quadricep muscle and adipose tissue. Evidence for significant lipolysis in skeletal muscle. J Clin Invest 96:370–377.
2. Gutierrez A, Anderstam B, Alvestrand A. 1999. Amino acid concentration in the interstitium of human skeletal muscle: a microdialysis study. Eur J Clin Invest 29:947–952.
3. Gilanyi M, Ikrenyi C, Fekete J, Ikrenyi K, Kovach AG. 1988. Ion concentrations in subcutaneous interstitial fluid: measured versus expected values. Am J Physiol 255:F513-519.
4. Fogh-Andersen N, Altura BM, Altura BT, Siggaard-Andersen O. 1995. Composition of interstitial fluid. Clin Chem 41:1522–1525.
